# Delayed Diagnosis of Duodenal Metastasis From Primary Lung Adenocarcinoma: A Case Report

**DOI:** 10.7759/cureus.45235

**Published:** 2023-09-14

**Authors:** Kim Abbegail T Aldecoa, Megan Frame, Alexander M Satei, Judie Goodman

**Affiliations:** 1 Internal Medicine, Trinity Health Oakland Hospital/Wayne State University Program, Pontiac, USA; 2 Internal Medicine, Ross University School of Medicine, Bridgetown, BRB; 3 Hematology and Oncology, Trinity Health Oakland Hospital/Wayne State University Program, Pontiac, USA; 4 Diagnostic Radiology, Trinity Health Oakland Hospital/Wayne State University Program, Pontiac, USA; 5 Hematology and Oncology, Trinity Health IHA Medical Group, Hematology Oncology - Oakland Campus, Pontiac, USA

**Keywords:** non-small cell lung cancer, gastrointestinal metastasis from lung cancer, duodenal metastasis from lung cancer, rare metastases, metastatic non-small cell lung cancer, adenocarcinoma, duodenal mass, lung cancer with gastrointestinal metastasis, gastrointestinal metastasis, lung cancer

## Abstract

The incidence of lung cancer metastasizing to the duodenum is rare, and its clinical presentation is still not fully understood due to its low frequency. It can be asymptomatic or present symptomatically in various ways. Here, we present the case of a 63-year-old female with an unusual case of duodenal metastasis from pulmonary adenocarcinoma, presenting with a new-onset seizure complicated by a fracture from a post-ictal fall. The diagnosis of anemia secondary to duodenal metastasis from lung cancer was delayed due to this sequence of events. The patient was ultimately found to have a circumferential mass in the third portion of the duodenum on esophagogastroduodenoscopy, which was found to be consistent with metastatic pulmonary adenocarcinoma on pathological examination.

## Introduction

Lung cancer is the leading cause of cancer-related deaths globally. In the United States, lung cancer accounts for the highest percentage of cancer deaths in both males and females, followed by prostate (males) and breast (females) cancer. The two main subtypes of lung cancer are small-cell lung carcinoma (SCLC, 15%) and non-small-cell lung carcinoma (NSCLC, 85%), which comprises squamous cell carcinoma, adenocarcinoma, and large-cell carcinoma [1]. Adenocarcinoma is the most common type of NSCLC and is the slowest growing of all types. NSCLC commonly metastasizes to the bone (34%), brain (28%), adrenal glands (17%), liver (13%), and extrathoracic lymph nodes (9%) [2,3].

Gastrointestinal metastasis is extremely rare [4,5]. Its rarity can be attributed to its presentation, which is usually asymptomatic. However, if symptomatic, clinical manifestations vary from abdominal pain, melena, vomiting, symptomatic anemia, upper gastrointestinal bleeding, and changes in bowel habits [6]. Our report involves a case of duodenal metastasis secondary to lung adenocarcinoma in a female patient with a rare initial presentation of seizure. The study aims to demonstrate the challenges of identifying duodenal metastasis in lung cancer patients due to a multitude of possible clinical presentations.

## Case presentation

A 63-year-old Caucasian woman, who has been diagnosed with Stage IV adenocarcinoma on the seventh cycle of pembrolizumab, presented to the emergency department with a new onset of generalized tonic-clonic seizure resulting in loss of consciousness and a post-ictal fall. She was noted to be confused after the seizure but did not experience urinary or stool incontinence. She had also been experiencing worsening fatigue in recent weeks. A review of the systems was negative for abdominal pain, shortness of breath, chest pain, melena, or hematochezia.

**Figure 1:**
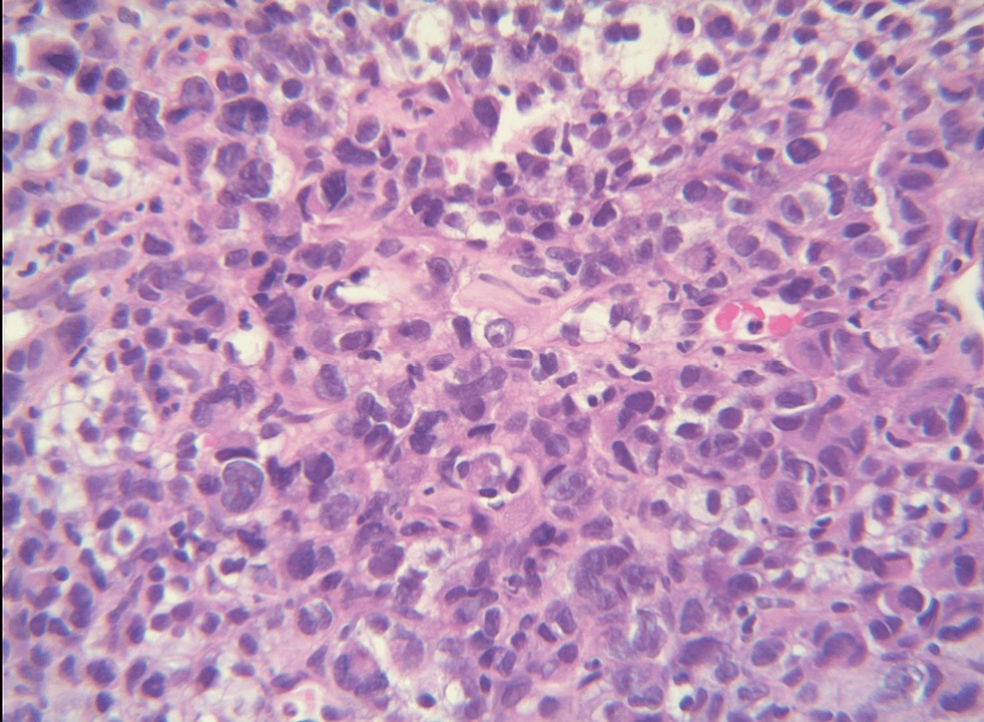
Lung biopsy consistent with poorly differentiated adenocarcinoma at 40× magnification.

**Figure 2:**
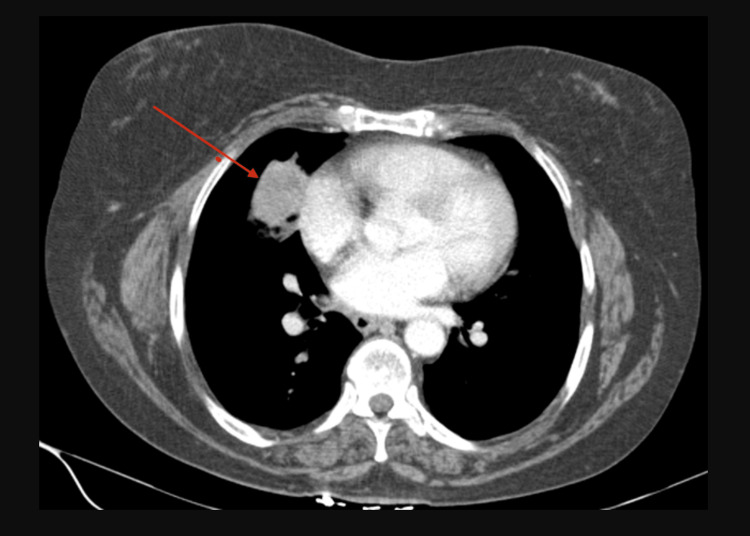
Contrast-enhanced computed tomography of the chest in axial view performed for initial staging showing a heterogeneously enhancing, lobulated, non-calcified right middle lobe mass closely abutting the cardiac border and measuring 4.0 × 3.1 × 2.3 cm. There is mild central low attenuation suggestive of necrosis.

The patient was diagnosed with metastatic lung adenocarcinoma 10 months before presentation. At that time, biopsy results showed a poorly differentiated NSCLC (Figure 1) positive for thyroid transcription factor-1 (TTF-1) and p40. On initial diagnosis, surgery was not feasible due to the patient’s poor lung function and the proximity of the mass to the heart border (Figure 2). The patient subsequently received chest radiation for limited disease but unfortunately demonstrated aggressive disease shortly after completion. A computed tomography (CT) scan of the chest, abdomen, and pelvis demonstrated increasing size of the previous lung mass and distant metastasis in the bilateral adrenal glands, with no metastasis elsewhere. The patient was subsequently clinically staged as IV (T3N2M1). Due to the high programmed death-ligand 1 level (tumor proportion score 70%) found with genetic profiling, the patient was started with single-agent pembrolizumab seven months ago. A follow-up CT scan of the chest, abdomen, and pelvis four months ago showed improvement of previously identified lesions with a decrease in the size of the previously identified lung mass and adrenal lesions.

**Figure 3:**
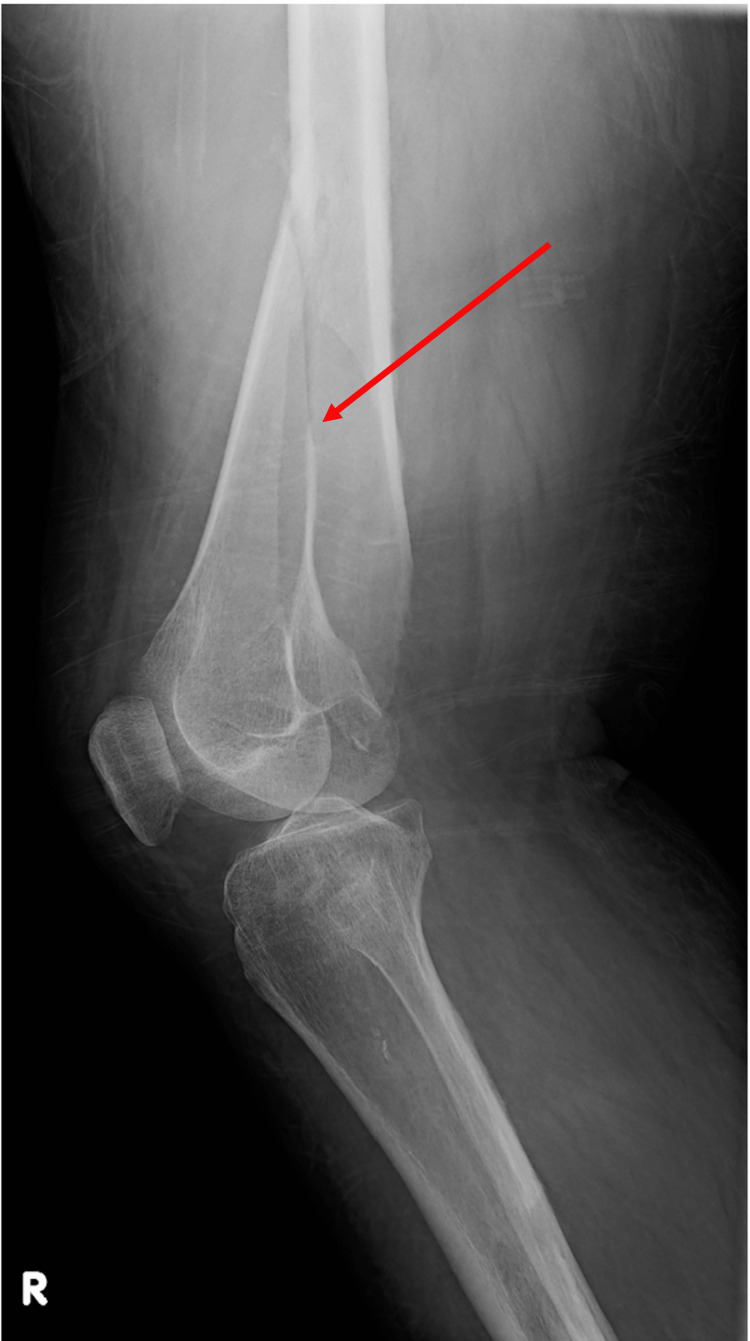
Lateral radiograph of the right knee demonstrating an obliquely oriented, displaced, mildly angulated acute fracture (red arrow) of the right distal femoral metadiaphysis with overriding of the fracture fragments.

**Figure 4:**
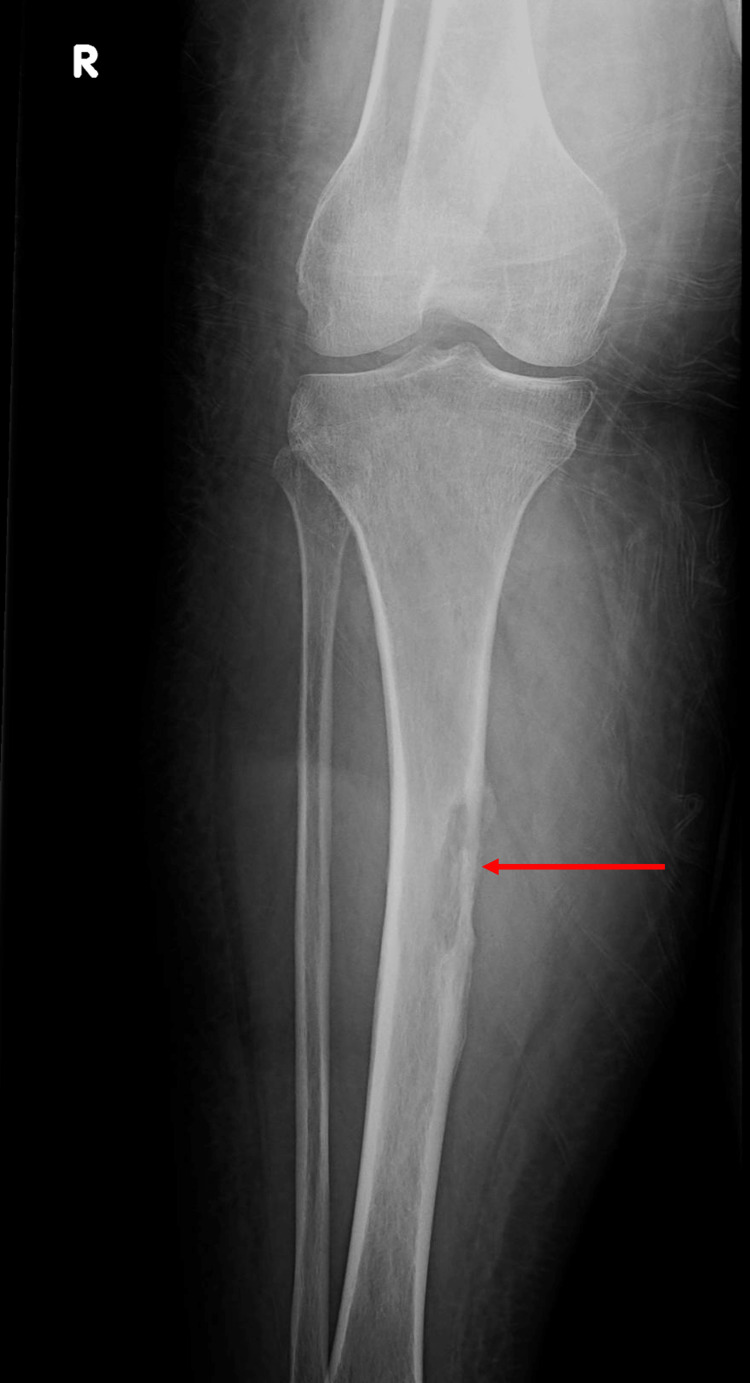
Anteroposterior radiograph of the right knee demonstrating an incidentally found lucent lesion with adjacent periosteal reaction within the proximal tibial diaphysis (red arrow).

**Figure 5:**
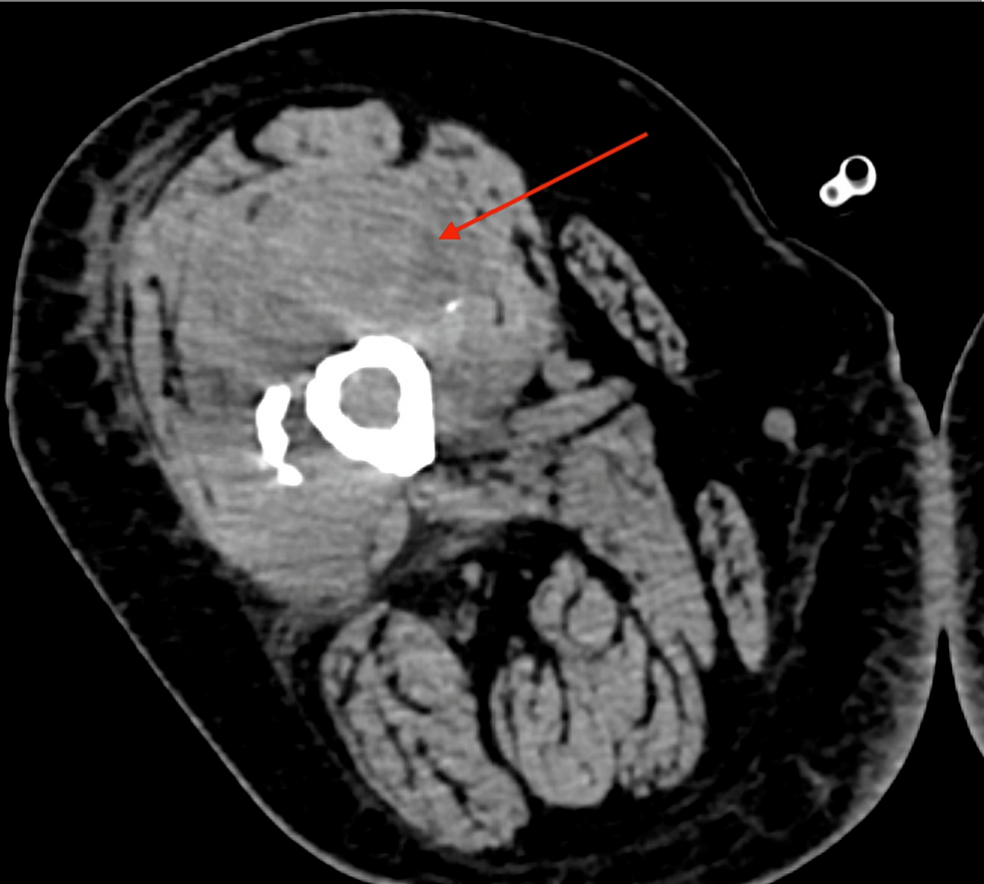
Contrast-enhanced computed tomography in axial view taken after plain film radiography demonstrated a fracture showing a mixed attenuating fluid collection anterior to the femoral fracture, suggestive of hematoma formation (red arrow).

On examination in the emergency, vital signs were stable. Physical examination was significant for right thigh swelling and a shortened externally rotated right lower extremity. Her labs were significant for hemoglobin of 5.5 g/dL, requiring two units of packed red blood cells (PRBCs). Plain film radiography of the right knee revealed an acute oblique fracture of the right distal femur (Figure 3) and an incidental lucent lesion within the right proximal tibial diaphysis (Figure 4). CT of the lower extremity also showed a significant acute intramuscular hematoma within the right vastus intermedius muscle (Figure 5). Magnetic resonance imaging (MRI) of the brain was ordered, but the patient expressed fear of undergoing an MRI and instead opted to have a CT scan of the head. CT of the head was negative for brain metastasis or any acute abnormalities. Open reduction and internal fixation of the right femur was performed. The patient was discharged after surgery with a hemoglobin of 7.2 g/dL. There was no recurrence of seizures during hospitalization. Due to negative structural lesions found on imaging and the patient’s first episode of seizure activity, the patient was not discharged on any anti-seizure medications.

**Figure 6:**
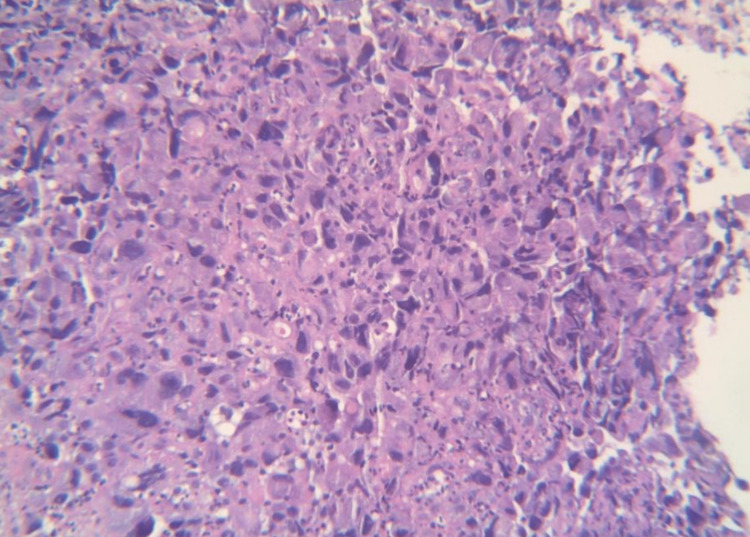
Duodenal biopsy consistent with adenocarcinoma.

**Figure 7:**
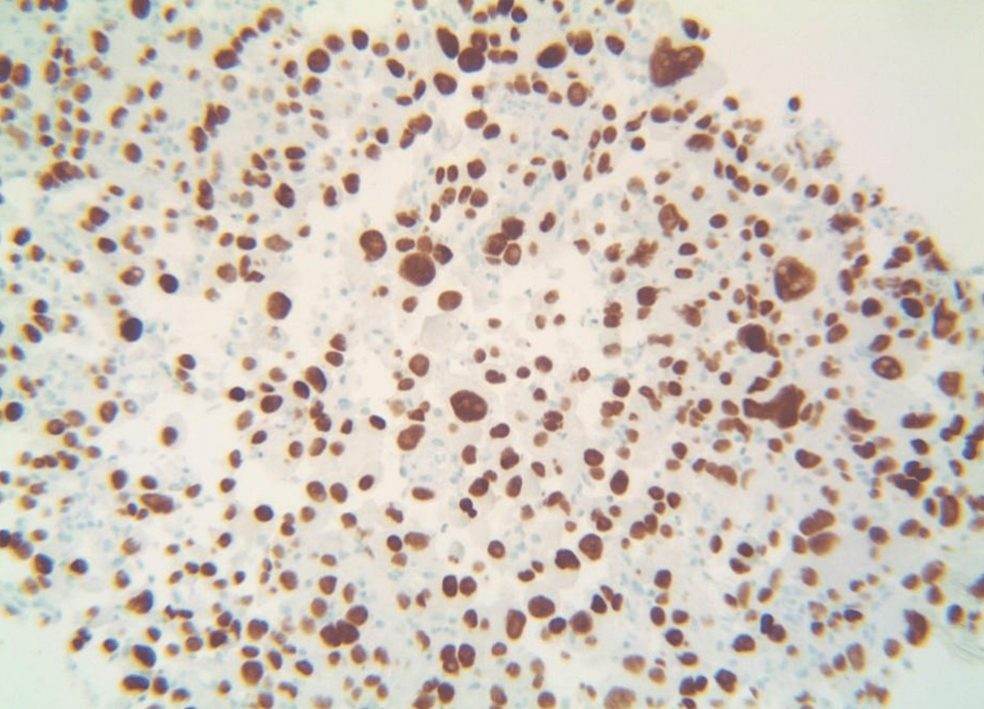
Duodenal biopsy with positive thyroid transcription factor-1 immunohistochemical stain showing adenocarcinoma consistent with metastasis from lung primary.

The patient returned to the emergency department two weeks after the surgery complaining of lightheadedness accompanied by intermittent abdominal pain and new-onset melena. Physical examination was significant for mild generalized tenderness to palpation of the abdomen but no guarding or rebound tenderness. The rectal examination was negative for gross blood, but the fecal occult blood test with guaiac showed trace results. Laboratory workup was significant for hemoglobin of 5.5 g/dL requiring two units of PRBCs (from 7.2 g/dL two weeks prior). Iron studies were remarkable for low iron of 28 µg/dL (normal: 50-212 µg/dL), iron saturation of 6% (normal: 20-55%), and total iron binding capacity of 455 µg/dL (normal: 250-450 µg/dL). Reticulocyte count was 7% (normal: 0.5-1.5%). Gastroenterology was consulted, and the patient underwent elective esophagogastroduodenoscopy (EGD). EGD revealed a large infiltrative, intraluminal, circumferential mass with stigmata of recent bleeding in the third portion of the duodenum, which spanned about 5 cm. A biopsy of the duodenal lesion revealed cytomorphological and immunohistochemical findings (Figures 6, 7) similar to previously identified poorly differentiated NSCLC, hence favoring a metastatic process. Due to the patient’s rapid course of progression and poor functional status, the duodenal mass was deemed unresectable and the patient was elected for palliative care management only. Unfortunately, the patient passed away two months after the diagnosis of gastrointestinal metastasis from lung cancer.

## Discussion

Gastrointestinal metastasis of primary lung cancer is uncommon. Clinical studies have shown a prevalence rate of only 0.2-1.7% [4-6], while autopsy studies have reported a slightly higher rate of 4.7-14% [4,5,7]. Among the documented cases of gastrointestinal metastasis from lung cancer, the small intestine is the most affected location due to its abundant blood supply. Specifically, the jejunum is the most frequent site of involvement (50.9%), followed by the ileum (33.3%) and duodenum (15.8%) [4].

The most common histologic subtype of lung cancer with gastrointestinal metastasis varies among literature with the following occurrences: adenocarcinoma (27.6-31.6%), squamous cell carcinoma (28.1%-28.5%), and large-cell carcinoma (20.9-24.6%). Among these histologic subtypes, large-cell carcinoma exhibited the highest propensity risk for gastrointestinal metastasis, while adenocarcinoma exhibited the least. However, due to the high prevalence of both adenocarcinoma and squamous cell carcinoma, most metastases to the small intestines are found to have originated from these two histologic subtypes. The most common pathway for metastasis is the hematogenous route via the spinal veins, but the lymphatic route through the retroperitoneum or mesentery has also been documented [4,8].

The low clinical incidence of duodenal metastasis may be attributed to their presentation, which is usually asymptomatic. However, when symptomatic, it can present with symptoms such as abdominal pain, melena, symptomatic anemia, upper gastrointestinal bleeding, and changes in bowel movements depending on the site of duodenal involvement [3,6,8]. Early recognition of these symptoms and prompt intervention can prevent further clinical deterioration of the patient. In a large study conducted by Liu et al., most patients with symptomatic presentation had complications of gastrointestinal metastasis, including perforation (42%), hemorrhage (24.6%), obstruction (20.4%), and intussusception. Small bowel metastasis has a relatively worse prognosis as it often leads to perforation [4,8]. Table 1 shows other presentations of adenocarcinoma that have a similar pathologic diagnosis to our patient’s case [9-16].

**Table 1:** Previously published cases of duodenal metastasis from non-small-cell lung carcinoma, adenocarcinoma type. GI: gastrointestinal; SMA: superior mesenteric artery; GERD: gastroesophageal reflux disease

Reference	Age (years)/Sex	Symptoms	Time of diagnosis	Duodenum location	Treatment	Status
Steinhart et al., 1991 [9]	55/M	Upper GI bleeding	Approximately three years after the primary lung cancer diagnosis	The third part of duodenum invading SMA	Conservative (including vasopressin)	Deceased, massive GI bleed
Cremon et al., 2002 [10]	66/M	Upper GI bleed	Eight months after treatment for primary lung cancer	-	-	Deceased, massive GI bleed
Kastakou et al., 2007 [11]	61/M	Melena, weight loss, hemoptysis	At the time of diagnosis of primary lung cancer. Initial presentation	The fourth part of the duodenum	Endoscopic resection, chemotherapy, blood transfusion, erythropoietin	Deceased, progressive lung disease
Linsen et al., 2015 [12]	68/M	Decreased exercise tolerance, iron deficiency anemia	At the time of diagnosis of lung cancer. Initial presentation	-	Chemotherapy	-
Alsaeed et al., 2015 [3]	52/M	Epigastric pain, melena, weight loss	At the time of diagnosis of primary lung cancer. Initial presentation	The fourth part of the duodenum	Resection, chemotherapy	-
Qasrawi et al., 2017 [14]	57/F	Left hip pain, anemia, melena	An unknown amount of years after primary lung cell cancer diagnosis	The second part of the duodenum	Chemotherapy	Deceased two weeks after the presentation
Kosasih et al., 2019 [13]	64/M	Melena, weakness, exertional dyspnea	At the time of diagnosis of primary lung cancer. Initial presentation	The second part of the duodenum	Conservative	Deceased
Kosasih et al., 2019 [13]	73/F	Weakness, exertional dyspnea, melena	At the time of diagnosis of primary lung cancer. Initial presentation	Distal duodenum	Chemotherapy and radiation	-
Tsai et al., 2020 [1]	54/M	GERD, superficial gastritis	Three years after the primary lung cancer diagnosis	-	-	-
O’Neil et al., 2020 [15]	56/M	Asymptomatic duodenal mets. Had a headache, dysarthria	At the time of diagnosis of lung cancer. Initial presentation	The second part of the duodenum	Chemotherapy	-
Kang et al., 2021 [16]	66/M	Melena, dizziness	Five months after lobectomy	The second part of duodenum + multiple small bowel metastasis	Total pancreatectomy, jejunal resection, ileal segment resection	Five months after the initial presentation, the patient is disease-free

Early detection, diagnosis, and treatment are crucial for improving patient outcomes in duodenal metastasis from primary lung cancer, which often goes unnoticed due to its benign clinical presentation, low incidence, and diagnostic challenges. Radiological imaging may manifest as an intraluminal polypoid mass or wall thickening with variable contrast enhancement patterns, but endoscopic evaluation with biopsy remains the most effective diagnostic method [6]. In our patient, the EGD confirmed the presence of a duodenal mass that was not detected by the CT scan of the abdomen. Simple diagnostic tests such as rectal exams and fecal occult blood tests can be performed early on to prevent any potential diagnoses from being missed. Positron emission tomography (PET) scans can be helpful in the detection of metastatic tumors of the duodenum in the area of the duodenal bulb or the periampullary region of the second part of the duodenum. However, the sensitivity and specificity of PET/CT to aid in diagnosing small bowel metastasis are still insufficient to date [6]. Immunohistochemistry is a valuable tool for diagnosis, with TTF-1 being an important marker of lung adenocarcinoma [1]. Our patient’s case displayed positive TTF results for both lung and duodenal biopsy samples.

There is no standard treatment for duodenal metastasis, but surgery, endoscopy, and chemoradiation therapy have been reported as options. Endoscopic resection can be a viable and safe option for a mass less than 1 cm in size, while surgical resection can be considered if the mass is larger than 1 cm in size, and even then, risks are involved [3,14]. The prognosis is generally poor with a median survival of only four months for patients with duodenal metastasis from primary lung cancer compared to bone metastasis with a median survival time of seven months and brain metastasis with a median survival time of three to six months [4, 14]. Overall, small bowel metastasis has a relatively shorter survival time than other gastrointestinal sites [5].

The patient’s seizure presentation and subsequent fracture and hematoma from a post-ictal fall delayed the diagnosis of active gastrointestinal bleeding from duodenal metastasis. Seizures can be a consequence of different mechanisms for cancer patients, such as primary or metastatic brain tumors (80%), adverse effects of treatments (surgery, radiation, and systemic therapies), electrolyte imbalance, or systemic infections in immunocompromised patients [17]. Lung cancer with brain metastasis can result in seizures, and brain MRI is typically used for diagnosis. However, in the case of our patient, an MRI was not done due to the patient’s expressed fear of the procedure. Instead, a CT scan of the head was performed, which did not reveal any mass lesions. Anemia can be another possible cause of the seizure as it has been linked to iron deficiency anemia and sickle cell anemia in certain groups [18]. The cause of the seizure in this patient is uncertain. However, this case highlights the importance of conducting a comprehensive physical examination and evaluation for new symptoms, such as anemia, which may be masked by other presentations.

## Conclusions

Gastrointestinal metastasis from lung cancer is rare, and its clinical characterization remains incomplete due to its low occurrences. It is most often asymptomatic. However, if symptomatic, it can present in various ways, such as seizures and anemia similar to what was observed in our patient. Active gastrointestinal bleeding from duodenal metastasis was missed initially because the anemia was attributed to significant hematoma from the post-ictal fall. This highlights the complexity of the presentation of duodenal metastasis from lung cancer. Therefore, a high index of suspicion and a thorough evaluation that begins with a complete physical examination is important. Cancer patients should be closely monitored for new symptoms to avoid delayed diagnosis and improve their quality of life with timely intervention. With better diagnostic techniques and advancements in targeted therapies, survival rates have improved, and duodenal metastasis may become more common in the future.

## References

[1] Tsai TJ, Chen TH, Tung CL (2020). Duodenum metastatic adenocarcinoma of lung origin: a case report. Adv Dig Med.

[2] Catalano M, Marini A, Ferrari K (2022). Gastric and colonic metastasis from NSCLC: a very unusual case report. Medicine (Baltimore).

[3] AlSaeed EF, Tunio MA, AlSayari K, AlDandan S, Riaz K (2015). Duodenal metastasis from lung adenocarcinoma: a rare cause of melena. Int J Surg Case Rep.

[4] Liu W, Zhou W, Qi WL, Ma YD, Xu YY (2015). Gastrointestinal hemorrhage due to ileal metastasis from primary lung cancer. World J Gastroenterol.

[5] Taira N, Kawabata T, Gabe A (2017). Analysis of gastrointestinal metastasis of primary lung cancer: Clinical characteristics and prognosis. Oncol Lett.

[6] Kim SY, Ha HK, Park SW (2009). Gastrointestinal metastasis from primary lung cancer: CT findings and clinicopathologic features. AJR Am J Roentgenol.

[7] Yoshimoto A, Kasahara K, Kawashima A (2006). Gastrointestinal metastases from primary lung cancer. Eur J Cancer.

[8] Hu Y, Feit N, Huang Y, Xu W, Zheng S, Li X (2018). Gastrointestinal metastasis of primary lung cancer: an analysis of 366 cases. Oncol Lett.

[9] Steinhart AH, Cohen LB, Hegele R, Saibil FG (1991). Upper gastrointestinal bleeding due to superior mesenteric artery to duodenum fistula: rare complication of metastatic lung carcinoma. Am J Gastroenterol.

[10] Cremon C, Barbara G, De Giorgio R (2002). Upper gastrointestinal bleeding due to duodenal metastasis from primary lung carcinoma. Dig Liver Dis.

[11] Kostakou C, Khaldi L, Flossos A, Kapsoritakis AN, Potamianos SP (2007). Melena: a rare complication of duodenal metastases from primary carcinoma of the lung. World J Gastroenterol.

[12] Linsen PV, Linsen VM, Buunk G, Arnold DE, Aerts JG (2015). Iron deficiency anemia as initial presentation of a non-small cell lung carcinoma: a case report. Respir Med Case Rep.

[13] Kosasih S, Muhammad Nawawi KN, Wong Z, Chia Hsin DC, Ban AY, Raja Ali RA (2019). Upper gastrointestinal bleed due to duodenal metastases of lung adenocarcinoma: report of two cases and review of literature. Case Rep Med.

[14] Qasrawi A, Tolentino A, Abu Ghanimeh M, Abughanimeh O, Albadarin S (2017). BRAF V600Q-mutated lung adenocarcinoma with duodenal metastasis and extreme leukocytosis. World J Clin Oncol.

[15] O'Neill RS, Duong T, Dionela W, Rogge C, Brungs D (2020). Pancreatitis and biliary obstruction secondary to duodenal metastasis from rapidly progressing lung adenocarcinoma treated with common bile duct stenting. Case Rep Oncol.

[16] Kang DK, Kang MK, Heo W, Hwang YH, Nam KH (2021). Multiple small bowel metastasis of primary non-small cell lung cancer. Clin Case Rep.

[17] Gonzalez Castro LN, Milligan TA (2020). Seizures in patients with cancer. Cancer.

[18] Padda J, Khalid K, Syam M (2021). Association of anemia with epilepsy and antiepileptic drugs. Cureus.

